# Detection tools for prediction and identification of adverse drug reactions in older patients: a systematic review and meta-analysis

**DOI:** 10.1038/s41598-022-17410-w

**Published:** 2022-08-01

**Authors:** Dewi Susanti Atmaja, Yulistiani, Suharjono, Elida Zairina

**Affiliations:** 1 Doctoral Program of Pharmaceutical Science, Faculty of Pharmacy, Universitas Airlangga, Surabaya, Indonesia; 2 Department of Pharmacy, Faculty of Health, Universitas Sari Mulia, Banjarmasin, Indonesia; 3 Department of Pharmacy Practice, Faculty of Pharmacy, Universitas Arlangga, Jalan Dokter Ir. Haji Soekarno, Mulyorejo, Surabaya, 60115 Jawa Timur Indonesia; 4 Innovative Pharmacy Practice and Integrated Outcome Research (INACORE) Group, Universitas Airlangga, Surabaya, Indonesia; 5 Center for Patient Safety Research, Universitas Airlangga, Surabaya, Indonesia

**Keywords:** Epidemiology, Outcomes research, Medical research, Risk factors, Geriatrics, Health services

## Abstract

Tools to accurately predict and detect adverse drug reactions (ADR) in elderly patients have not been developed. We aimed to identify and evaluate reports on tools that predict and detect ADR in elderly patients (≥ 60 years). In this review, we followed the Preferred Reporting Items for Systematic Reviews and Meta-Analysis (PRISMA) guidelines. Databases were searched until January 2022 using key terms “elderly,” “adverse drug reaction,” and “detection instruments.” Eighteen studies met the inclusion criteria, and they examined assorted interventions: STOPP/START version 1/2 (n = 10), Beers Criteria 2012 or 2015 (n = 4), Systematic Tool to Reduce Inappropriate Prescribing (STRIP) (n = 2), Tool to Reduce Inappropriate Medications (TRIM) (n = 1), Medication Risk Score (MERIS) (n = 1), Computerized alert systems (n = 1), and Norwegian General Practice-Nursing Home criteria (n = 1). The interventions affected the number of potential prescription omissions (OR, 0.50 [0.37–0.69]; *p* < 0.0001; four studies). No apparent reduction in the number of drug interactions within 2 months (OR, 0.84 [0.70–1.02]; *p* = 0.08; two studies) and mortality (OR, 0.92 [0.76–1.12]; *p* = 0.41; three studies) was observed. In conclusion, there is no definitive and validated assessment tool for detecting and predicting ADR in elderly patients. Thus, more research on refining existing tools or developing new ones is warranted.

## Introduction

Adverse drug reactions (ADR) are the main focus of the pharmacovigilance system, which is related to medication safety. According to the European Medicines Agency (EMA), ADR is defined as “a response to a drug product that is noxious and unwanted”. The definition is currently extended to allowable drug use, including unwanted drug reactions from off-label effects, poisoning, and medication errors^1^. Additionally, the US Food and Drug Administration defines ADRs as untoward medical events possibly caused by the use of drugs in humans, because there is a possibility that the drug causes adverse effects^2^. Increased life expectancy has led to an increase in the elderly population, who are more vulnerable to developing ADR as long as they use medications. Various factors can cause ADR, including aging-induced changes in physiology, resulting in conditions such as geriatric syndrome, comorbidity, and disease complexity. The elderly need to take medicines for maintaining their health and quality of life. They usually follow various drug regimens, which leads to the potential for drug interactions that can lead to ADR^3^.

When patients take more drugs, it is complicated to review the overall drug use which causes medication errors, potentially harmful interactions, and drug toxicity that can lead to hospitalization. The majority of drugs cause hospitalizations because of their side effects, including anticoagulants, antiplatelets, NSAIDs, opioids, and antihypertensives^4^. Identification of ADR should be evaluated objectively using a probability scale. Clinical medication reviews can help clinical pharmacists assess symptoms from patient interviews^5^. Causality assessment instruments can assist in providing data that health workers require to ensure the safety of patients using medications. The Naranjo algorithm and the assessment criteria established by the WHO Uppsala Monitoring Centre are the most commonly used tools for determining the causality of ADR events. However, both assessment instruments have advantages and disadvantages, and the Naranjo scale instrument cannot be used to evaluate more complex cases such as polypharmacy and multimorbidity^6^.

Because the physiology of elderly individuals differs from that of other populations (adults), it is necessary to select appropriate instruments for predicting and detecting ADR in the elderly to reduce and prevent ADR^6^. ADR causality assessment can contribute to pharmacovigilance through the evaluation of the risk profile; it can indicate the benefit of drugs and provide an early warning of potential ADR events. Causality instruments combined with trigger tools and health-worker competency can yield better results in ADR detection. A systematic review published in 2020 found 5 studies (meeting the studies' inclusion criteria) that suggested the high prevalence of adverse drug events (ADE) and ADR in older patients with dementia. Only one study documented ADE and has variability in ADR methods and definitions. Furthermore, the results may not apply to other populations and settings because of a limited number of wards^7^. Another systematic review published in 2014 found 4 studies that developed and validated the ADR prediction model for use in patients over 65 years old. However, assessment of the quality of studies was challenging due to poor reporting in the studies included in the review^8^. Therefore, there are no models that can be used as prediction tools for ADR^8^. Hence, we conducted a systematic review to recognize and analyze literature on tools/methods that can accurately predict and detect ADR in elderly patients (≥ 60 years). We (1) identified primary research related to the use or development of an ADR prediction and/or detection instrument used in elderly patients (≥ 60 years) with morbidity and polypharmacy, and (2) assessed the effectiveness of each instrument for ADR prediction and/or detection used in elderly patients (≥ 60 years) for preventing and reducing ADR.

## Results

### General characteristics of the studies

**Figure 1:**
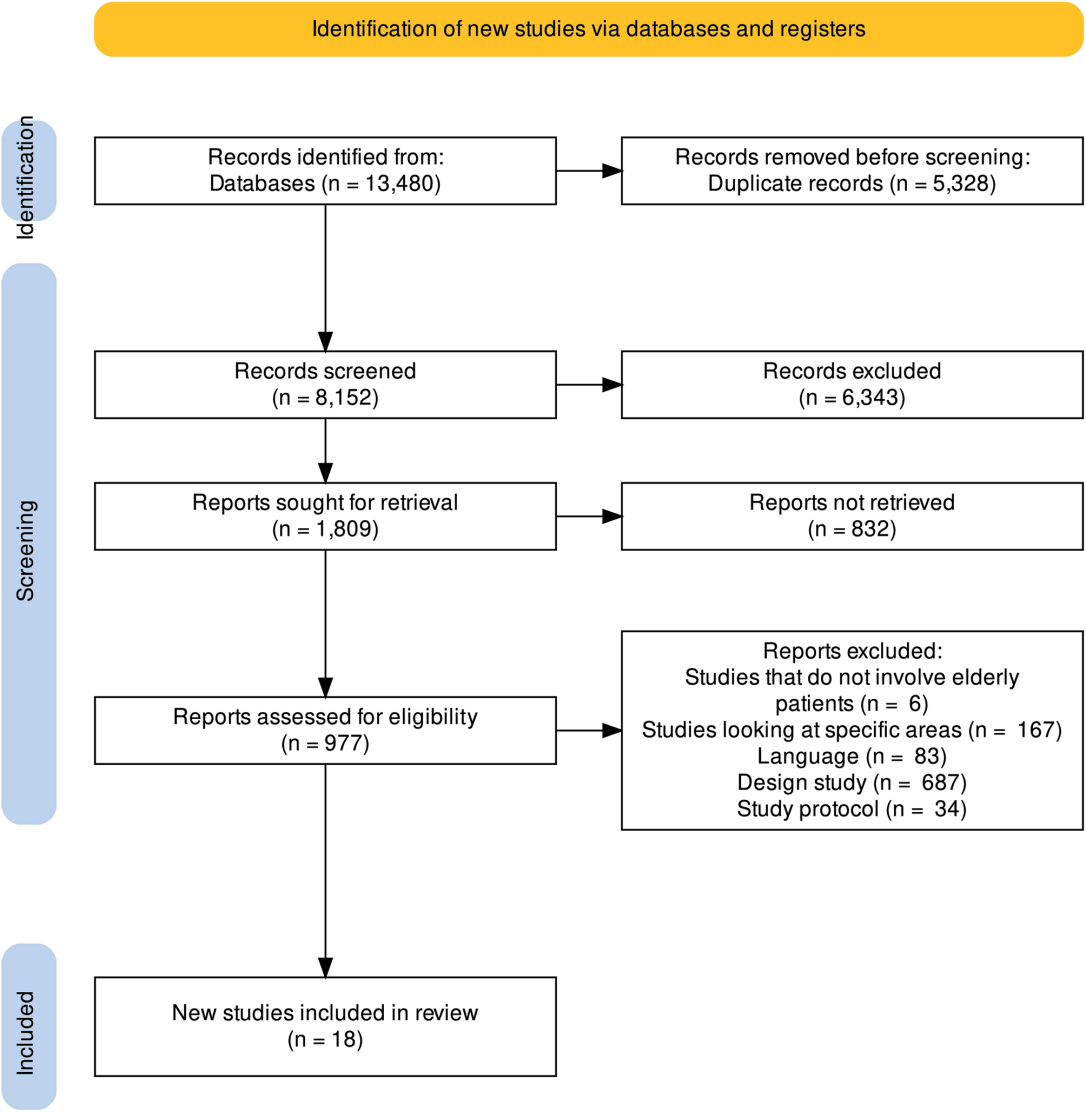
Flow diagram for study selection.

Of 13480 studies initially identified, 977 studies were retrieved and fully reviewed. We excluded 959 studies for the reasons outlined in the flow chart, based on the PRISMA guidelines (Fig. 1). Eighteen studies met the specific inclusion criteria at the end of the election process. Figure 1 represents the steps of the search and selection process. The typical studies included in this systematic review are presented in Supplementary Table S1. The included articles were published from 2011 to 2021; eleven of these studies were conducted in Europe, four in North America, two in Asia, and one in the Middle East. Regarding the design, ten studies were randomized controlled trials (RCTs); four, cluster randomized controlled trials (cRCTs); and four, controlled clinical trials (CCTs).

Most studies were conducted in a hospital setting and, according to the inclusion criteria, in elderly patients (seven studies in the inpatient ward; one, acute internal medicine ward; one, acute admissions unit; one, medical ward; one, geriatric inpatient ward; and one, acute geriatric ward). Four studies were conducted in the clinic (three studies in primary care and one in a geriatric outpatient clinic). Two studies were conducted in homes for elderly individuals. The median or mean age of respondents is specified in most of the studies, with the age of the respondents ranging from 72.4^9^ to 86.7 years^10^; however, two of the reports did not indicate the median or mean age of the respondents^11^, and one only specified the age of the respondents^12^. The number of respondents included in each study varied from 63^13^ to 81.905^14^. Only one report did not present the number of respondents; however, it presented the total number of homes for a mean of 35–48 elderly residents^11^. The study periods lasted up to 12 months.

### Screening tools used in the intervention

All included studies utilized simple to complex intervention tools related to ADR topics. Most tools used for screening potentially inappropriate medications (PIM) are STOPP/START version 1 or 2 (10 studies), Beers Criteria 2012 or 2015 (4 studies), Systematic Tool to Reduce Inappropriate Prescribing (STRIP) (2 studies), TRIM (1 study), MERIS (1 study), Norwegian General Practice-Nursing Home criteria (1 study), and Computerized alert systems (1 study).

### Risk of bias in included studies

**Figure 2:**
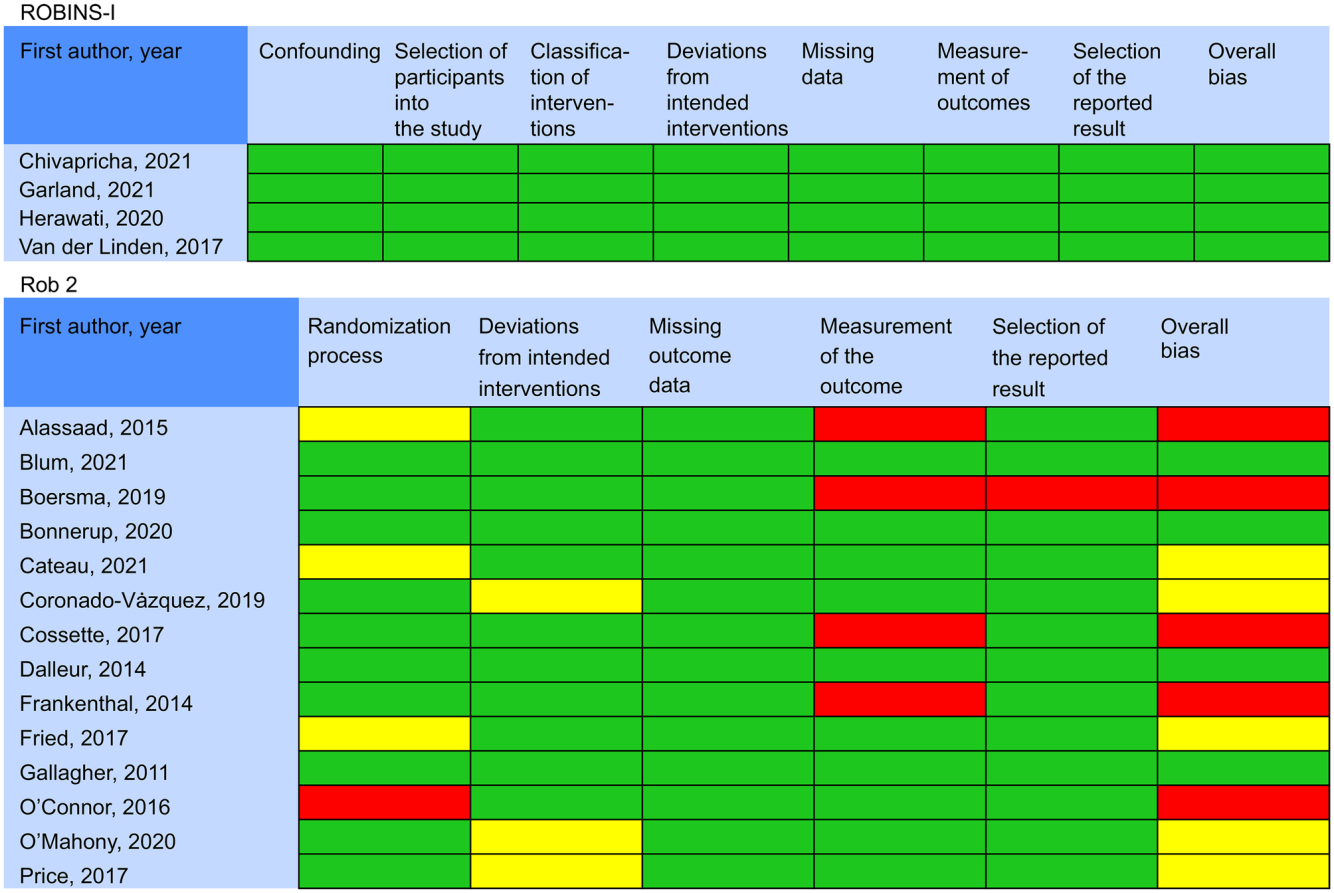
Risk of bias assessment.

Bias risk ranged from low to high across studies, with varying sources of bias. The risk of outcome bias was high in four studies, wherein the risk of randomization process bias was either high or cause for concern. The deviation from the intended intervention bias was a source of concern herein. The resulting risk bias was high in one study. Some of the included studies exhibited a low risk of bias (Fig. 2).

### Primary outcomes

*A number of predicted and prevented ADR or AE* was announced in three studies. Two studies showed no significant increase in the prevalence of falls with the STOPP/START screening intervention^15^. Nevertheless, one study showed that the prevalence was less during the six-month monitoring period, in the exposure group (5.8% vs. 8.4%; *p* = 0.332)^16^. One study that utilized the STRIP intervention demonstrated no significant difference in the number of first preventable drug-related hospital admissions (*p* = 0.49; Hazard Ratio was 0.89 [0.63–1.25])^17^.

Overall, the intervention with STOPP/START or STRIP probably leads to no change in the number of predicted and prevented ADR or AE in elderly patients (moderate evidence quality). The evidence was downgraded because of high or unclear ROB across multiple domains (− 1).

*A number of reductions in ADR or AE* was reported in three studies. Two studies showed a significant difference in the number of discontinued medications or medications subjected to dose reduction. One study with pharmacist and physician intervention using CAS demonstrated a higher rate of drug discontinuation and dosage reduction in the intervention group versus the comparison group two days after the alert (+ 30.0%) and hospital repatriation (+ 20.8%) but no significant difference in the number of in-hospital deaths. However, the analysis showed an almost 4% dwindle in the intervention group versus the comparison group^18^. One study with pharmacist intervention showed that 18% more medicines were stopped or subjected to dose reduction in the exposure group, considering the number of medications administered upon admission (comparison vs. exposure: median [IQR] was 0.32 [0.21–0.49] vs. 0.50 [0.42–0.63]; p < 0.001) and identified by the “Rationalization of Home Medication by an Adjusted STOPP in Older Patients” (RASP) List (comparison vs. intervention: median [IQR] was 1 [1, 2] vs. 2 [1–4]; p = 0.003)^19^. One study with STOPP/START screening exhibited a significant reduction in the mean number of falls in the exposure group within the monitoring period (p = 0.006); however, no significant reduction in the mean number of hospitalizations was observed in the exposure group (p = 0.40)^15^.

Overall, the CAS-based pharmacist–physician intervention, pharmacist intervention, and STOPP/START screening may lead to little or no effect in reducing ADR or AE in elderly patients (low evidence quality). The evidence was downgraded because of high or unclear ROB across multiple domains (− 1) and impreciseness because of low event numbers (− 1).

*A number of reductions in exposure to polypharmacy* was observed in three studies and was significant in the exposure group. Two studies with STOPP/START screening intervention showed a 14.6% reduction in polypharmacy in the exposure group at discharge^16^ and a reduction in the mean number of medications in the exposure group throughout the monitoring period (*p* < 0.001) and in the comparison and exposure groups at the end of the study (*p* < 0.001), these results are from (*p* < 0.001)^15^. A study that utilized the PEPS model intervention demonstrated a more significant decrease in the mean number of regular medications in the exposure group, from 8.96 at the starting point to 6.88 at 12 months, compared with that in the comparison group (from 9.85–8.87) ([DID] 1.1; *p* < 0.001). Additionally, the mean number of medications was significantly decreased when testing all medications^20^.

Overall, the intervention with STOPP/START screening and PEPS is likely to affect the number of reducing polypharmacy in elderly patients (low evidence quality). Because of high or unclear ROB across multiple domains (− 1) and impreciseness because of low event numbers (− 1). The evidence was downgraded.

**Figure 3:**
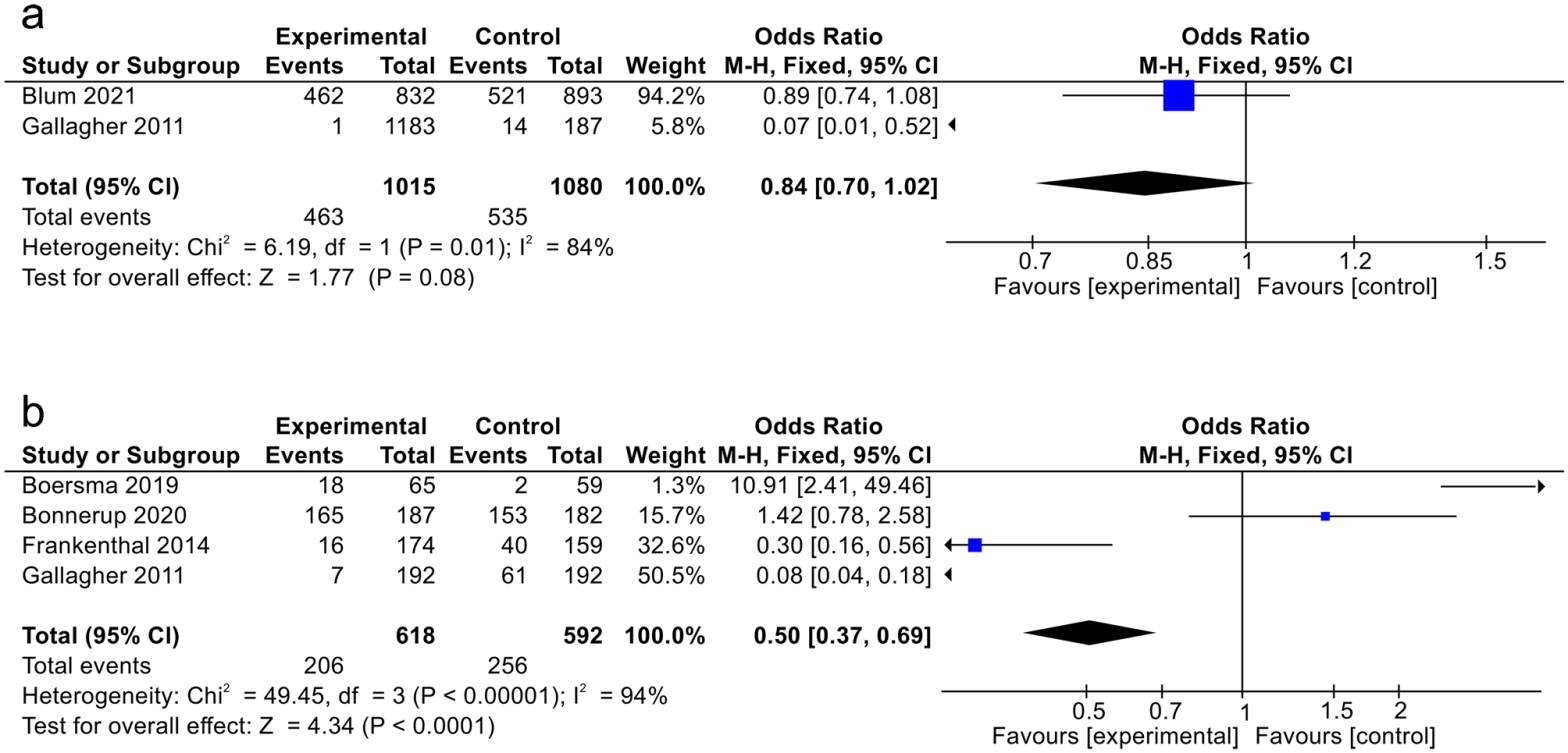
(**a**) “Meta-analysis” of the effect of interventions on A: number of reducing drug interactions within 2 months; (**b**) number of potential prescription omission (PPO).

*A reduction in the number of drug interactions* was analyzed in two studies. One study with STOPP/START screening intervention showed significantly reduced drug-drug interaction in the exposure group at discharge and throughout the monitoring; a reduction was observed in the number of drug-disease interactions from admission to discharge (5% in the comparison group and 11.2% in the exposure group)^16^. One study with STRIP intervention showed no significant difference in the reduction of drug-drug interactions within 2 months between the exposure and comparison groups (55.5% vs. 58.3%; *p* = 0.31; odds ratio was 0.87 [0.67–1.14])^17^. Meta-analysis of the number of reduced drug interactions within two months investigated as an outcome measure in 2095 elderly patients showed no significant difference between the exposure and comparison groups (odds ratio was 0.84 [0.70–1.02]; *p* = 0.08) with the heterogeneity of *I*^2^ = 84% (Fig. 3A).

Overall, the intervention with STOPP/START screening may influence the reduction in the number of drug interactions; however, STRIP may not affect the reduction in the number of drug interactions in elderly patients (moderate evidence quality). The evidence was downgraded because of heterogeneity in reporting of the outcome (–1).

*The number of inappropriate medications* was evaluated in ten studies, which showed mixed effects. Eight studies demonstrated a significant number of inappropriate medications or reduction of PIM or *potentially inappropriate prescription* (PIP). One study with STRIP intervention demonstrated significant changes in the number of implemented PIM between the exposure and comparison groups (46.2% vs. 15.3%, *p* < 0.005; odds ratio was 0.14 [0.07–0.57]^21^. One study delivered geriatric pharmacy specialist intervention with pharmaceutical care; it showed a significant decrease in the prevalence of PIM upon discharge from the hospital in the exposure group (21.3% and 43.3%, *p* < 0.05); additionally, the number of PIM was lesser in the exposure group than in the comparison group (21.3% vs. 40.9%, *p* = 0.036). However, no significant difference in prevalence on admission was observed when compared to that during hospitalization in either group^22^. One study with Intervention Inpatient Geriatric Consultation Team (IGCT) using STOPP criteria recommendations showed a significant reduction in PIM discontinued at hospital admission compared with that at discharge in the exposure and comparison groups (39.7% vs. 19.3%; *p* = 0.013; odds ratio was 2.75 [1.22–26.24]), but no significant subtraction at the patient level for the prevalence of PIM among the exposure and comparison group (23.1% vs. 16.1%; *p* = 0.454; odds ratio was 1.5 [0.49–4.89])^23^. One study with STOPP/START screening intervention showed a significant reduction in PIPs in the exposure group at six months of monitoring (*p* < 0.001) and during 6 to 12 months of monitoring (*p* < 0.001) compared to that at the starting point^15^. One study with TRIM intervention showed that the number of medication reconciliation faults was significantly higher in the exposure group than in the comparison group (48.4% vs. 14.3%; *p* < 0.001)^12^. One study with PEPS model medication (*p* = 0.002) and all medications (regular and as needed) (*p* < 0.001) between the exposure and comparison groups showed a significant reduction, but no significant prevalence ≥ 1 PIM in regular medication (*p* = 0.37) and all medication (*p* = 0.12)^20^. One study with pharmacist intervention showed a significant difference in PIMs identified by RASP between the exposure and comparison groups (median [IQR] 0.5 [0–1] vs. 2 [1–3]; *p* < 0.001)^19^. One study with shared-decision-making intervention reported a significant difference in the mean number of inappropriate medications withdrawn post-monitoring between the exposure and comparison groups (mean difference was 0.34 [0.01–0.66]; *p* = 0.04); however, no significant difference was observed in the group of respondents whose medication was altered after 6 months of monitoring (odds ratio was 2.8 [1.3–6.1]; *p* = 0.008)^24^. Two studies showed no significant difference in the number of inappropriate medications related to PIM or PIP. One study with QC-Mode intervention showed no significant difference in the number of PIM galenic units (regression coefficient was − 0.014[− 0.038 + 0.010]; *p* = 0.240) and reduction in the number of PIM defined daily dose resident (regression coefficient was − 0.183[− 0.392; + 0.025]; *p* = 0.083) between the exposure and comparison groups^11^. One study with STOPP-guideline-based intervention showed no significant alteration in the PIP rate between the exposure and comparison groups (*p* = 0.80)^14^.

Overall, most of the interventions affected the number of inappropriate medications; only a few showed no effect (very low evidence quality). The evidence was downgraded because of high or unclear ROB across multiple domains (− 1), heterogeneity in reporting of the outcome (− 1), and impreciseness because of low event numbers (− 1).

*The number of potential prescription omissions* was observed in four studies, all related to prescribing. Three studies showed a significant reduction in potential prescribing omissions (PPOs). Two studies with STOPP/START screening intervention showed a significant reduction in PPOs. One of these studies showed a significant reduction in the exposure group at six months of monitoring (*p* < 0.001) but no significant reduction during 6 to 12 months of monitoring (*p* > 0.99); nevertheless, the prevalence of PPOs tended to drop. This study also showed a significant number of PPOs among the two groups at 12 months of monitoring (*p* < 0.001)^15^. Another study demonstrated a reduction in the incidence of incorrectly prescribed doses in the exposure group at discharge, the prevalence of prescribing omissions, and under-prescribing (1.2%; 31.6%; 31.4%)^16^. One study with STRIP intervention showed a significant difference in the number of implemented PPO between the exposure and comparison groups (26.2% vs. 3.4%; *p* < 0.001); however, the difference in the number of doses is not significant between the exposure and comparison groups (4.6% vs. 0.0%; *p* = 0.1) because of suboptimal dosage^21^. One study with stratified medication review intervention showed no significant difference in the number of prescribing errors between the exposure and comparison groups, based on events per patient (mean was 0.88 [0.67–1.09] vs. 0.84 [0.67–1.01]; *p* = 0.86) and events per drug (mean was 0.11 [0.08–0.14] vs. 0.13 [0.09–0.16]; *p* = 0.65); the significantly greater number of prescribing errors were discovered in the high-risk group^9^. Meta-analysis of the number of potential prescription omissions investigated as an outcome measure in 1210 elderly patients indicated a significant difference between the exposure group and comparison group (odds ratio was 0.50 [0.37–0.69]; *p* < 0.0001) with the heterogeneity of *I*^2^ = 94% (Fig. 3B).

Overall, the intervention with STOPP/START screening and STRIP may affect the number of potential prescription omissions in elderly patients (very low evidence quality). The evidence was downgraded because of high or unclear ROB across multiple domains (− 1), heterogeneity in reporting of the outcome (− 1), and impreciseness because the confidence interval includes the potential for significant harm or benefit (− 1).

*The number of ADRs identified* was determined in eleven studies, which appeared to exhibit a mixed impact. Eight studies indicated no significant difference in the number of identified ADRs related to its general incidence, falls, rehospitalization, and mortality because of ADR. Two studies with STOPP/START intervention showed no significant difference between the exposure and comparison groups with respect to the frequency of hospital readmissions over 6 months of monitoring (67 vs. 64; *p* = 0.691)^16^, mean number of hospitalizations (*p* = 0.10), and mean number of falls (*p* = 0.28)^15^. Two studies with STRIP intervention showed no significant difference between the exposure and comparison groups with respect to mortality (13.1% vs. 12.1%; *p* = 0.859^21^ and 4.3% vs. 5.2%; *p* = 0.38; hazard ratio was 0.81 [0.51–1.29]) and first drug-related hospital admission (21.9% vs. 22.4%; *p* = 0.62; hazard ratio was 0.95 [0.77–1.17])^17^. One study with QC-Mode intervention showed no significant difference in the number of falls (*p* = 0.575) and mortality (*p* = 0.06)^11^. One study with CAS-based pharmacist–physician intervention showed no significant difference in readmissions at 30 days post-discharge (15.9% vs. 21.9%; *p* = 0.3)^18^. One study with SENATOR intervention showed no significant difference in ADR incidence between the exposure and comparison groups at the primary endpoint (24.5% vs. 24.8%; odds ratio was 0.98 [0.77–1.24]; *p* = 0.88), at the secondary endpoint (S1) (33.7% vs. 36.7%; odds ratio was 0.87 [0.70–1.08]; *p* = 0.20), (S2) (21.2% vs. 22.9%; odds ratio was 0.91 [0.71—1.15]; *p* = 0.42), and post-hoc; additionally, no significant difference was observed for rehospitalization rate (36.2% vs. 34.9%; odds ratio was 1.05 [0.84–1.32]; *p* = 0.66)^25^. One study with pharmacy intervention announced no significant difference between the exposure and comparison groups with respect to mortality (*p* = 1.000; *p* = 1.000) and number of falls (*p* = 0.742; *p* = 0.954) during hospitalization and after discharge and with respect to rehospitalization after discharge (*p* = 0.629)^19^. Three studies showed a significant number of identified ADRs related to general ADR/ADE incidence, GerontoNet score, rehospitalization, and mortality. Two studies with STOPP/START intervention showed a significant difference in the incidence of ADR or ADE between the exposure and comparison groups (11.7% vs. 21.0%; odds ratio was 0.50 [0.33–0.75]; *p* = 0.001^26^ and 3 vs. 13; *p* = 0.017^27^) and for GerontoNet score at the time of step outside (mean = 3.3(2.3) vs. 5.2(2.1); *p* = 0.003)^27^. One study with comprehensive clinical pharmacist intervention showed a significant difference in the number of identified rehospitalizations (mean was 1.15 [1.01–1.32]) and mortality rate (mean was 0.40 [0.33–0.48])^10^.

Overall, most of the interventions may not affect the number of identified ADRs; only a few showed any effect (low evidence quality). The evidence was downgraded because of high or unclear ROB across multiple domains (− 1) and heterogeneity in reporting of the outcome (− 1).

### Secondary outcomes

*MAI score* was announced in two studies with STOPP/START screening exposure and appeared to be significantly different between the exposure and comparison groups following discharge (mean 2.97(2.25) vs. 9.94(6.14); *p* < 0.001)^13^; the scores decreased from 10 (3–16.25) upon admission to 3 (1–6) at the time of discharge (*T* = 447, *P* < 0.001, *r* =  − 0.52) and continued to decrease throughout the monitoring period (*X*^*2*^_F_ = 226.312, *P* < 0.001)^16^.

Overall, the intervention with STOPP/START screening is likely to affect MAI scores in elderly patients (moderate evidence quality). Because of the low event numbers, the evidence was downgraded because of impreciseness (− 1).

*AOU* was announced in one study. The number required to identify with STOPP/START criteria to give a betterment in the AOU at the time of hospital step outside for the exposure group was 4.7 (3.4–7.5)^16^.

Overall, the STOPP/START screening intervention may affect AOU in elderly patients (moderate evidence quality). The evidence was downgraded because of the impreciseness (− 1) of the low event numbers.

**Figure 4:**
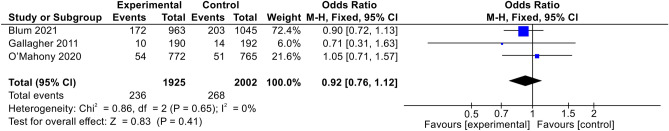
A “meta-analysis” of the effect of interventions on mortality (all-cause).

*Mortality (all-cause)* was determined in three studies; no significant difference was observed between the exposure and comparison groups. One study with STRIP intervention showed no significant difference in mortality between the exposure and comparison groups (19.4% vs. 17.9%; hazard ratio was 0.90 [0.7–1.13]; *p* = 0.37)^17^. In one study with STOPP/START screening intervention, the prevalence of mortality attributed to any cause was less in the exposure group than in the comparison group over the 6-month monitoring period; nevertheless, the difference was not significant (5.3% vs. 7.3%; *p* = 0.414)^16^. One study with SENATOR intervention showed no significant mortality rate within 30 days between the exposure and comparison groups (7.2% vs. 7.1%; odds ratio was 1.05 [0.70–1.57]; *p* = 0.81)^25^. Meta-analysis of the number of potential prescription omissions investigated as an outcome measure in 3927 elderly patients indicated no significant difference between the exposure and comparison groups (odds ratio was 0.92 [0.76–1.12]; *p* = 0.41) with the heterogeneity of *I*^2^ = 0% (Fig. 4).

Overall, the STRIP, STOPP/START screening, and SENATOR interventions are likely to reduce mortality (all causes) in elderly patients (moderate evidence quality). The evidence was downgraded because of inconsistency with the confidence interval, which includes the possibility of significant critical harm or benefit (− 1).

*Length of Stay* was determined in five studies. Two studies with STOPP/START screening intervention showed no significant difference in the duration of stay between the exposure and comparison groups (median [IQR] 8 [5–14] vs. 8.5 [5–15.75], p=0.471^16^; median [IQR] 8 [4–14] vs. 8 [4–14]). The median length of stay was significantly longer among respondents who developed ADR relative to those who did not (median [IQR] 10 [6–14, 17, 19, 20] vs. 7 [4–14]; *p* < 0.001)^26^. One study with CAS-based pharmacist–physician intervention showed no significant difference in length of stay between the exposure and comparison groups (median [IQR] 10 [6–14, 17, 19, 20, 25, 27] vs. 9.5 [5–14, 17, 19, 20, 24, 25, 27, 30]; *p* = 0.9)^18^. One study with SENATOR intervention showed no significant difference in length of stay between the exposure and comparison groups (median [IQR] 6 [3–10] vs. 6 [3–11])^25^. One study with STOPP/START screening intervention showed a significant difference in length of stay between the exposure and comparison groups (mean 7.6 [3,0] vs. 14.2 [10,0]; *p* = 0.011)^13^.

Overall, most of the interventions may not affect the length of stay; only a few showed any effect (very low evidence quality). The evidence was downgraded because of high or unclear ROB across multiple domains (− 2) and impreciseness because of low event numbers (− 1).

*Quality of Life* was determined in five studies. Four studies with EQ-5D-3L measurement showed no significant difference in the quality of life between the exposure and comparison groups (0.358[SD 0.016] vs. 0.294 [SD 0.018])^19^ at day 90 of the monitoring period (*p* = 0.73) compared with that at the initiation of monitoring (*p* = 0.65)^9^; the quality of life was not significantly different after discharge compared with that at the starting point for both groups (DM was 0.14[0.11–0.18]; *p* < 0.001). However, no significant difference was observed in the quality of life at discharge and at the starting point between the exposure and comparison groups (DM[95%CI] =  − 0.02[− 0.10–0.06]; *p* = 0.5942)^25^. Significantly superior quality of life was observed after one year in the exposure group compared to that in the comparison group (17.8% vs. 19.1%; mean difference was 2.29[0.31–4.26]; *p* = 0.02)^17^. One study with an SF-12 questionnaire showed no significant difference in the quality of life between the exposure and comparison groups with respect to the mean scores for the physical ( *p* = 0.09) and mental components (*p* = 0.70) after one year of monitoring^15^.

Overall, the interventions may have little or no impact on the quality of life in elderly patients (moderate evidence quality). The evidence was downgraded because of heterogeneity in reporting of the outcome (− 1).

## Discussion

Eighteen studies included in this systematic review provided significantly varying results, according to the synthesis of quantitative analysis of their outcomes and meta-analysis suitable only for three outcomes in this review. Previous systematic reviews have evaluated screening tools used by clinical pharmacists for the identification of drug-related problems among elderly patients^27^. In this study, we included all tools utilized by the health workers for detecting ADR, and this is the first study to perform such an evaluation using the indicated criteria. We additionally provide a synthesis of narratives from the findings. The interventions targeted over 8000 elderly individuals; however, the exact number of elderly individuals was poorly reported in two studies^11,14^. Most of the trials were conducted in hospitals; however, some were conducted in clinics and homes for the elderly. Currently, the output of this systematic review cannot support the use of any instrument or tool intended to predict or detect ADR for the prevention and reduction of ADR. Only three studies demonstrated the prediction or prevention of ADR using STOPP/START screening and STRIP as an instrument; however, the results are not significant. Nevertheless, STOPP/START resulted in a lower prevalence of ADR in the exposure group than in the comparison group (5.8% vs. 8.4%; *p* = 0.332)^16^. Most of the instruments such as STOPP/START successfully identified ADR related to general ADR/ADE incidence, GerontoNet score, rehospitalization, and mortality. The results for the exposure and comparison groups (11.7% vs. 21.0%; odds ratio was 0.50 [0.33–0.75]; *p* = 0.001^26^ and 3 vs. 13; *p* = 0.017^27^) and GerontoNet score at the time of step outside (mean = 3.3(2.3) vs. 5.2(2.1); *p* = 0.003)^27^. Furthermore, comprehensive clinical pharmacist intervention also successfully identified the number of rehospitalizations (mean was 1.15 [1.01–1.32]) and the mortality rate (mean was 0.40 [0.33–0.48])^10^. Most of the instruments successfully identified the number of inappropriate medications and reduction of PIM or PIP. The STRIP intervention successfully changed the number of implemented PIM between the exposure and comparison groups (46.2% vs. 15.3%, *p* < 0.005; odds ratio was 0.14 [0.07–0.57]^21^. The geriatric pharmacy specialist intervention with pharmaceutical care also decreased the prevalence of PIM upon discharge from the hospital in the exposure group (21.3% and 43.3%, *p* < 0.05); additionally, the number of PIM was lesser in the exposure relative to the comparison group (21.3% vs. 40.9%, *p* = 0.036)^22^. The START/STOPP criteria successfully reduced the PIM discontinuation at hospital admission relative to that at discharge between the exposure and comparison groups (39.7% vs. 19.3%; *p* = 0.013; odds ratio was 2.75 [1.22–26.24])^23^. Reduction in PIPs in the exposure group at six months of monitoring (*p* < 0.001) and during 6 to 12 months of monitoring (*p* < 0.001) as compared to that at the starting point was recorded^15^. The TRIM intervention showed that the number of medication reconciliation faults was higher in the exposure group than in the comparison group (48.4% vs. 14.3%; *p* < 0.001)^12^. Reduced PIM results in a decrease in the PEPS model (*p* = 0.002) and all medications (regular and as needed) (*p* < 0.001) between the exposure and comparison groups^20^. The pharmacist intervention successfully identified PIMs by RASP between the exposure and comparison groups (median [IQR] 0.5 [0–1] vs. 2 [1–3]; *p* < 0.001)^19^. The shared-decision-making intervention also exhibited a difference in the mean number of inappropriate medications withdrawn post-monitoring between the exposure and comparison groups (mean difference was 0.34 [0.01–0.66]; *p* = 0.04)^24^. However, they were associated with low- or very low-quality evidence, which implies that we cannot recommend any of the currently published instruments for predicting or detecting ADR in elderly patients. A previous systematic review also evaluated the interventions based on health outcomes in reducing the incidence of ADEs in elderly patients in primary care settings, but the results of the interventions showed no significant benefit in terms of health outcomes such as hospitalization, emergency department visits, mortality, and quality of life. The intervention of prescription or review of medications by health workers was the most commonly used approach by the pharmacists^28^.

Our meta-analysis identified that the interventions affect the number of PPOs; however, heterogeneity was high among the included studies. This heterogeneity could be attributed to variability in the interventions and methodological diversity. However, our findings must be interpreted with caution. Although a substantial number of studies were included in this review, there remains the need for further high-quality evidence for an instrument that adequately predicts or detects ADR. None of the studies included in this review provided external validation that affords conviction that a particular instrument’s prognostic capability is credible across different populations and settings.

## Limitations

This systematic review has several possible limitations. The search strategies for identifying relevant studies included the use of “adverse drug reaction prediction and/or detection instruments” as a fundamental term of search. While this was expected to be applicable because the systematic review aimed to prioritize patients subjected to adverse drug reaction prediction and/or detection instruments, other research that predicted or detected ADR in the elderly could have been excluded if the study methodology did not include instruments or tools. We used the PICOT framework to establish the search strategy, to reduce the possibility of "missing" related studies, and all four researchers thoroughly discussed the search terms to be used. Furthermore, language was also a limitation in the selection of included studies. It was difficult to conduct a meta-analysis for some studies, considering the type of methodologies and patient demographics, the outcomes, and the reporting of outputs. Another limitation is that some of the studies included in the meta-analysis exhibited significant statistical heterogeneity. Thus, the pooled effect estimates should be interpreted with caution. This variation could be attributed to intervention variability and methodological diversity. Therefore, future research should strive for greater agreement on defining and assessing preventability to reduce heterogeneity among studies and allow more precise estimates for future meta-analyses.

According to the findings of this systematic review, many tools have been developed for use in hospitals, clinics, and homes for the elderly for the prediction or detection of ADR in elderly patients. The complexity of the tools developed to date and the outcome measures and methods used for validating their performance vary. There is no definitive validated assessment tool for widespread use in older patients. The findings reveal that most instruments evaluated could identify ADR and inappropriate medications.

## Conclusion

According to the findings of this systematic review, many tools have been developed for use in hospitals, clinics, and homes for the prediction or detection of ADR in elderly patients. The complexity of the tools developed to date and the outcome measures and methods used for validating their performance vary substantially. There is no definitive validated assessment tool for widespread use for older patients. The findings reveal that most instruments evaluated could identify ADR and inappropriate medications.

## Methods

### Data sources and study selection

The authors developed a systematic search strategy. The database search was performed until 1 January 2022 without the application of date restrictions: EMBASE, MEDLINE (Ovid), CINAHL, Pubmed, Web of Science, and ProQuest.

The reviewers filtered all article titles based on the PICOT search strategy and tailored them to each database with no restrictions. Key search terms consisted of three main concepts: elderly with morbidity, adverse drug reaction prediction and/or detection instruments, polypharmacy, and an in-depth list of synonyms, given the variable terminology in the field.

If possible, the terminology of Medical Subject Headings was used (in OVID, EMBASE, and MEDLINE); for databases not using the terminology of Medical Subject Headings, keywords were used (CINAHL, Pubmed, Web of Science, and ProQuest). The search strategy was developed by the authors in collaboration with an experienced librarian. The complete details of the search strategies have been provided in the supplementary material.

We used the Endnote and Rayyan programs to classify the results and excluded the same articles^29^. The review of titles and screening of the article abstracts used in this study were performed by two independent reviewers. The previous stage involved screening of the full-text review of the article by two independent reviewers; if an agreement was not reached, a third reviewer was consulted. Each search stage records all exclusion criteria for creating a flow diagram as stated by the components heeled in “Preferred Reporting Items for Systematic Reviews and Meta-Analyses” (PRISMA)^30,31^. The selection process is repeated independently for both purposes.

### Inclusion criteria

In this review, we included CCT- or RCT-based original research/studies published in English and Indonesian that enrolled elderly patients (≥ 60 years old) with morbidity and polypharmacy as respondents, and hospitals, homes for elderly individuals, and communities with public and private healthcare systems as research settings.

### Exclusion criteria

We excluded the following studies from the review: literature reviews, systematic reviews, meta-analyses, discussion articles, conference proceedings, summary articles, editorials, studies on patients with COVID-19, and studies investigating specific parameters (e.g., HbA1c control, blood glucose, or dementia) that did not involve elderly patients (≥ 60 years). Additionally, we excluded research protocols whose results had not been published or were not available in the database and for which only abstracts were available for analysis.

### Data extraction, synthesis, and analysis

The full text was assessed independently by two reviewers (DA and EZ), and data were extracted. The data extraction forms were designed considering the study’s two objectives, and the data was entered into tables in the Excel software. We pulled all research articles that present the use of an instrument for predicting and/or detecting ADR in elderly patients, based on the first objective. Subsequently, based on the second objective, we extracted all research articles that present the evaluation results of using an instrument for predicting and/or detecting ADR in elderly patients, as indicated by the number of predicted and prevented ADR or *adverse events* (AE), polypharmacy, drug interactions, number of inappropriate medications, and number of potential prescription omissions (PPO).

“RoB 2 and ROBINS-I” were handled as tools for appraising the risk of bias^32,33^. RoB 2 is used for RCT studies and ROBINS-I for CCT studies. To appraise the evidence quality, we used GRADE, and two researchers conducted all of these procedures to validate the obtained data^34^.

The reviewers performed data analyses using the Review Manager (RevMan) software^35^, version 5.4 after data extraction based on the objectives of this systematic review. The odds ratios (OR) for dichotomous outcomes were calculated alongside the 95% confidence intervals (CIs). To determine the heterogeneity of the studies, I^2^ was calculated. The summary of grouped data included research methods, population characteristics, settings, and research outcomes. We studied these results in depth to draw conclusions that answer the research question posed in this systematic review.

The protocol of this systematic review is registered in PROSPERO (CRD42021240621).

## Supplementary Information

Supplementary Information.

## Data Availability

All data generated or analyzed during this study are included in this published article.
