# Supplementary material for: Detection tools for prediction and identification of adverse drug reactions in older patients: a systematic review and meta-analysis
Source: Sci Rep. 2022 Aug 1;12:13189. doi: 10.1038/s41598-022-17410-w (PMC9341414; doi:10.1038/s41598-022-17410-w)
Supplement: Supplementary file 1 — Supplementary Information. [file 41598_2022_17410_MOESM1_ESM.docx]

**Supplementary Table S1-** Description of studies included in the systematic review

| **Researcher and year** | **Location** | **Study Setting** | **Number of participants (n)** | **Mean Age (year)** | **Study design** | **Study objective** | **Primary Outcome** |
| --- | --- | --- | --- | --- | --- | --- | --- |
| Gallagher et al. [16] | Ireland | Inpatient wards, Cork University Hospital | 382 patients | 74.5 (intervention)  77 (comparison) | Randomized controlled trial | To filter the medications of hospitalized older patients using STOPP/START criteria and allocate results to accompanying medical team that result in significant and sustained advances in prescribing appropriateness compared to usual hospital pharmaceutical care. | 1. “MAI” scores at admission and discharge and over the 6-month monitoring 2. AOU index at admission and discharge and over the 6-month monitoring period, determining the number of patients with at least one prescribing omission |
| Blum et al. [17] | Four European countries (Netherlands, Switzerland, Republic of Ireland, and Belgium) | Inpatient wards, University-based hospitals | 2008 patients | 79 | Cluster randomized controlled trial | To analyze the effect of optimizing drug treatment on elderly patients with multimorbidity and polypharmacy admitted to hospital. | The first confirmed drug-related hospital admission after discharge following the index hospital admission within 12 months of enrollment |
| Alassaad et al. [10] | Sweden | Uppsala University hospital (Two acute internal medicine wards) | 368 patients | 86.7 | Randomized controlled trial | To arrange and internally validate a risk “80+ score”, for revisits and mortality, integrating aspects of pharmacotherapy and to compare the discriminatory ability of the score with that of three validated tools for evaluating inappropriate prescribing. | Number of rehospitalizations or deaths during the 12 months after discharge from hospital. |
| Boersma et al. [21] | Netherlands | University Medical Centre Utrecht (Geriatric outpatient clinic) | 34 residents  124 patients | 77.8 (intervention)  79.0 (comparison) | Cluster randomized controlled trial | To analyze the STRIP Assistant recommendation on appropriate prescribing and mortality in a preoperative setting | The number of implemented medication changes by residents |
| Bonnerup et al. [9] | Denmark | Aarhus University Hospital  (Acute Admissions Unit) | 375 patients | 72.4 (intervention)  72.8 (comparison) | Randomized controlled trial | To evaluate medication reviews according to MERIS and to examine the algorithm in a large patient population | The number of prescribing errors during hospitalization of the respondents (except errors in discharge summaries) |
| Cateau et al. [11] | Switzerland | The cantons of Fribourg and Vaud (Nursing home) | 56 NH | N/A | Randomized controlled trial | To decrease the number of PIMs | The number of PIM (galenic units and daily dose per midpoint resident and per day (DDD/res)) |
| Chivapricha et al. [22] | Thailand | Medical ward, a tertiary university hospital in Bangkok | 187 patients | 74.0 (intervention)  74.7 (comparison) | A prospective, quasi-experimental study | To establish PIM prevalence on admission and compare changes following discharge, with comprehensive care by a ward pharmacist or without a geriatric pharmacy specialist. | The number of PIMs on admission |
| Cossette et al. [18] | Québec (Canada) | Centre Hospitalier Universitaire de Sherbrooke (Inpatient wards) | 254 patients | 81.5 (intervention)  80.5 (comparison) | Pragmatic single-site randomized controlled trial | 1. The effect of CAS-based pharmacist–physician intervention model compared to usual clinical care in PIMs 2. An assessment of the length of stay, in-hospital deaths, and emergency room visits and readmissions within 30 days of discharge | The number of discontinued drugs or drugs with a dosage reduction among the total number of medications for which the pharmacist indicated a drug cessation or dosage reduction |
| Dalleur et al. [23] | Belgium | Cliniques uni- versitaires Saint-Luc teaching hospital in Brussels (Inpatient wards) | 146 patients | 84 (intervention)  86 (comparison) | Randomized controlled trial | To analyze effect of STOPP criteria interventions on discontinuation of PIMs in elderly inpatient discharged from a hospital. | The number of PIMs discontinued at hospital admission compared with that at discharge (according to the discharge letter) |
| Frankenthal et al. [15] | Israel | A chronic care geriatric facility in central Israel (Inpatient wards) | 359 patients | 82.7 | Parallel-group randomized trial | To assess effect of a STOPP/START intervention on clinical and economic outcomes. | The mean number of falls and hospitalizations, quality of life as assessed using the Medical Outcomes Study 12-item Short-Form Health Survey (SF-12), and medication costs |
| Fried et al. [12] | United States | Veterans Affairs (VA) Connecticut Healthcare System (Primary care clinics) | 128 patients | Intervention  <70 = 27 (42.2%) 70–79 = 31 (48.4%) ≥80 = 6 (9.4%)  Comparison  <70 = 25 (39.1%) 70–79 = 26 (40.6%) ≥80 = 13 (20.3%) | Randomized controlled trial | To evaluate effect of TRIM, a web tool linking an electronic health record (EHR) to a clinical decision support system, on medication communication and prescribing | Patient perception regarding participation in their care and patient–clinician medication-associated communication |
| Garland et al. [20] | Québec (Canada) | LTCFs in Québec (Canada) | 691 patients | 84.1 (intervention)  85.8 (comparison) | A pragmatic quasi-experimental study with a control group | To evaluate the effect of a new pharmaceutical care model intervention on polypharmacy and PIM use in long-term care facilities (LTCFs) | The number of patients with polypharmacy and PIMs |
| Herawati et al. [13] | Indonesia | Sanglah General Hospital, Indonesia (Geriatric Inpatient Ward) | 63 patients | 72.5 (intervention)  67.8 (comparison) | A non-randomized controlled trial | To analyze STOPP/START intervention to advance the Adapted Medication Appropriateness Index (MAI) and to reduce the risk of ADRs (GerontoNet score) and length of stay | “MAI” score, GerontoNet Score, and LOS |
| O’connor et al. [26] | Ireland | Tertiary referral hospital in southern Ireland (Inpatient wards) | 732 patients | 80 (intervention)  78 (comparison) | Single-blind cluster randomized controlled trial | To reduce incidents of hospital-acquired adverse drug reactions (ADRs), 28-day medication costs, and median length of hospital stay in older adults admitted with acute illness by STOPP/START intervention | 1. The number of ADR in the participants encountering one or more ADR during the index hospitalization 2. Median length of stay and 28-day total medication cost |
| O’mahony et al. [25] | Ireland, Scotland, Spain, Italy, Belgium and Iceland | Inpatient wards,  six European medical centers | 1537 patients | 78 | Pragmatic prospective randomized open-label, blinded endpoint (PROBE) controlled trial | To evaluate effect of clinical decision support systems (CDSS) interventions for generated medication to reduce ADR incidence | ADR incidence, mortality rate, rehospitalization rate, and quality of life (EQ5D-3L) |
| Price et al. [14] | Canada | Primary care in British Columbia | 8 clinics  28 physicians  81905 patients | N/A | A mixed-method, pragmatic, cluster, randomized control trial | To analyze impact of STOPP prescribing criteria, implemented in a routinely used primary care Electronic Medical Record (EMR), for PIP rates in community (non-academic) primary care practices | The number of PIPs between the exposure and comparison groups before the intervention compared with the variance after the intervention period |
| Van der Linden et al. [19] | Belgium | University hospital in Flanders, Belgium (Three acute geriatric wards) | 172 patients | 84.5 | A monocentric, prospective controlled trial | To assess the effect of a pharmacist intervention, consisting of implementation of “RASP” list and a pharmacist-led medication review on polypharmacy, the quality of prescribing, and clinical outcome in elderly inpatients. | 1. The combination endpoint of drug cessation and dose reduction of drugs taken upon admission 2. “RASP” identified PIMs, the quality of life, and number of Emergency Department visits registered up to 3 months after discharge |
| Coronado-Vazquez et al., [24] | Spain | Primary care, health centers in Aragon and Andalusia, Spain | 22 physicians and nurses  122 patients | 78.9 (intervention)  79.9 (comparison) | A randomized, multicenter quasi-experimental study | To analyze a decision-making support tool for deciding medication appropriateness in patients with one or more chronic diseases (hypertension, dyslipidemia, and/or diabetes) and polypharmacy in the primary care setting | The variance among groups in terms of medication appropriateness, defined as drug recall or substitution in accordance with STOPP/Beers criteria and drug initiation in accordance with START criteria |

**Supplementary Table S2-** Summary of findings

| **Outcomes** | **Impact** | | | | | | | | | | | **Number of studies** | **Quality of the evidence (GRADE)** |
| --- | --- | --- | --- | --- | --- | --- | --- | --- | --- | --- | --- | --- | --- |
| Number of prediction and prevention adverse drug reaction (ADR) or adverse events (AE) | All studies showed no significant difference in number of prediction and prevention of adverse drug reactions or adverse events. One study showed that the prevalence of falls was lesser in the exposure group than in the comparison group over 6 months of monitoring, but the difference was not statistically significant. Another one study announced no significant difference in prevalence of falls between the exposure and comparison groups. One study showed no significant difference for first preventable-related hospital admissions between exposure and comparison groups. | | | | | | | | | | | 3 | Moderate^a^ |
| Number of reducing ADR or AE | All studies showed a significant difference in number of reduced adverse drug reactions or adverse events. Two studies showed significant difference in discontinued or reduced drugs between the exposure and comparison group, but one of these studies showed no significant difference for in-hospital deaths between the exposure and comparison groups. Another study showed a significant decrease in mean number of falls in the exposure group within the monitoring period, but no significant decrease in the mean number of hospitalizations in the intervention group. | | | | | | | | | | | 3 | Low^b^ |
| Number of reducing polypharmacy | All studies announced a significant reduction in polypharmacy at discharge and during the monitoring period compared with that at admission in the intervention group. | | | | | | | | | | | 3 | Low^c^ |
| Number of reducing drug interaction within two months | **No. of patients** | | | | | | | **Effect** | | | | 2 | Moderate^d^ |
|  | **Exposure group** | | | **Comparison Group** | | | **Relative (95% CI)** | | | | **Absolute (95% CI)** |  |  |
|  | 463/1015 (45.16%) | | | 535/1080 (49.5%) | | | **OR 0.84**  (0.70 to 1.02) | | | | **43 fewer per 1000** (from 88 fewer to 5 more) |  |  |
| Number of inappropriate medications | Five studies showed significant difference in PIM between exposure and comparison groups, which four studies compare the section on admission and step outside, and one study compares section until 12 months monitoring. Within the intervention group, three studies showed no significant difference in PIM between the exposure and comparison groups. One study found a significant difference in the prevalence of PIM on discharge from hospitalization between the exposure and comparison groups. Another study found no significant difference in PIM prevalence reduction between the exposure and comparison groups. One study showed significant reduction in PIPs for the exposure group during the intervention period, but another study showed no significant PIP rate between the exposure and comparison groups. | | | | | | | | | | | 10 | Low^e^ |
| Number of potential prescription omission | **No. of patients** | | | | | **Effect** | | | | | | 4 | Very Low^f^ |
|  | **Exposure group** | | **Comparison Group** | | **Relative (95% CI)** | | | | **Absolute (95% CI)** | | |  |  |
|  | 206/618 (33.3%) | | 256/592 (43.2%) | | **OR** 0.50 (0.37 to 0.69) | | | | **157 fewer per 1000** (from 213 fewer to 88 fewer) | | |  |  |
| Number of identification ADR | Three studies showed a significant difference in identification of adverse drug reactions between the exposure and comparison groups, which one study appeared a significant in rehospitalization and mortality, and another one study appeared a significant in GerontoNet score. The result for six studies in rehospitalization, four studies in mortality rate, and three studies in falls is no significant among the exposure and comparison group. | | | | | | | | | | | 11 | Low^g^ |
| MAI score | All studies showed a significant difference in MAI Score between the exposure and comparison groups. One study showed that improvement in MAI score was less at discharge and during the six-month monitoring period. | | | | | | | | | | | 2 | Moderate^h^ |
| AOU | A significant reduction in AOU in the intervention group at discharge and within the monitoring period. Improvement in AOU at discharge is 4.7. | | | | | | | | | | | 1 | Moderate^i^ |
| Mortality (all-cause) | **No. of patients** | | | | **Effect** | | | | | | | 3 | Moderate^j^ |
|  | **Exposure group** | **Comparison Group** | | | **Relative (95% CI)** | | | | | **Absolute (95% CI)** | |  |  |
|  | 236/1925 (12.3%) | 268/2002 (13.4%) | | | **OR** 0.92 (0.76 to 1.12) | | | | | **9 fewer per 1000** (from 29 fewer to 14 more) | |  |  |
| Length of Stay | Four studies showed no significant difference in the length of stay between the exposure and comparison groups, but one of these studies showed a significant difference in length of stay between participants who encountered an ADR and those who did not. In another study, length of stay was significantly different between the exposure and comparison groups during the study period. | | | | | | | | | | | 5 | Very Low^k^ |
| Quality of Life | Within the study period, one study showed a significant difference in quality of life between the exposure and comparison groups. Four studies found no significant in quality of life between the exposure and comparison groups. Overall, EQ-5D was used as a tool in the studies; only one study used SF-12 as a tool. | | | | | | | | | | | 5 | Moderate^d^ |

Footnotes

a. One mark was deducted as long as high risk of bias in one study related to inappropriate method for measuring the outcome.

b. One mark was deducted as long as high risk of bias in one study related to inappropriate methods for measuring the outcome. One mark was deducted because of impreciseness with low event numbers in all studies, and confidence interval could not be measured in two studies.

c. One mark was deducted as long as high risk of bias in one study related to inappropriate method for measuring the outcome. One mark deducted because of impreciseness with low event numbers in two studies, and confidence interval could not be measured in all studies.

d. One mark was deducted because of heterogeneity in reporting of the outcome.

e. One mark was deducted because of a high or unclear risk of bias across studies, with five of ten studies having more than 50% of the domains as high or unclear ROB. One mark was deducted because of heterogeneity in reporting of the outcome. One mark deducted because of impreciseness with low event numbers in nine studies, and confidence interval could not be measured in six studies.

f. One mark was deducted because of a high or unclear risk of bias across studies, with two of four studies having blinding of outcome assessment in one study and inappropriate method for measuring the outcome in one study. One mark was deducted because of heterogeneity in reporting of the outcome. One mark deducted because of impreciseness with confidence interval included the potential for important harm or benefit.

g. One mark was deducted because of a high or unclear risk of bias across studies, with seven of eleven studies having more than 50% of the domains as high or unclear ROB. One mark was deducted because of heterogeneity in reporting the outcome.

h. One mark was deducted because of impreciseness with low event numbers, and confidence interval could not be measured in all studies.

i. One mark was deducted because of impreciseness as only one study was identified.

j. One mark deducted because of impreciseness with confidence interval included the potential for important harm or benefit.

k. One mark was deducted as long as high or unclear risk of bias across studies. Three of five studies had blinding of outcome assessment in one study and differences in starting point characteristics in one study, and no information about deviations from intended interventions.

**Supplementary file:** Search strategies

PUBMED/WEB OF SCIENCE

<https://pubmed.ncbi.nlm.nih.gov/>

<https://www.webofscience.com/>

| 1 | Aged |
| --- | --- |
| 2 | Elderly |
| 3 | Geriatric |
| 4 | “older person” |
| 5 | “older people” |
| 6 | ADR prediction |
| 7 | ADR detection |
| 8 | Adverse drug reaction prediction |
| 9 | Adverse drug reaction detection |
| 10 | ADR prediction tool |
| 11 | ADR detection tool |
| 12 | Adverse drug reaction prediction tool |
| 13 | Adverse drug reaction detection tool |
| 14 | BEERS criteria |
| 15 | STOPP/START |
| 16 | Gerontonet ADR Risk Score |
| 17 | Brighton adverse drug reaction risk |
| 18 | World health organization Uppsala monitoring centre |
| 19 | Naranjo scale |
| 20 | PADR-EC Score |
| 21 | Adverse drug reaction risk in older persons scale |
| 22 | MERIS |
| 23 | MEDCOINS Score |
| 24 | Drug interaction probability scale |
| 25 | or/1-5 |
| 26 | 6 and 25 |
| 27 | 7 and 25 |
| 28 | 8 and 25 |
| 29 | 9 and 25 |
| 30 | 10 and 25 |
| 31 | 11 and 25 |
| 32 | 12 and 25 |
| 33 | 13 and 25 |
| 34 | 14 and 25 |
| 35 | 15 and 25 |
| 36 | 16 and 25 |
| 37 | 17 and 25 |
| 38 | 18 and 25 |
| 39 | 19 and 25 |
| 40 | 20 and 25 |
| 41 | 21 and 25 |
| 42 | 22 and 25 |
| 43 | 23 and 25 |
| 44 | 24 and 25 |
| 45 | Prevention |
| 46 | Reduce |
| 47 | Prevent |
| 48 | Avoid |
| 49 | Decrease |
| 50 | “cut down” |
| 51 | or/45-50 |
| 52 | 26 and 51 |
| 53 | 27 and 51 |
| 54 | 28 and 51 |
| 55 | 29 and 51 |
| 56 | 30 and 51 |
| 57 | 31 and 51 |
| 58 | 32 and 51 |
| 59 | 33 and 51 |
| 60 | 34 and 51 |
| 61 | 35 and 51 |
| 62 | 36 and 51 |
| 63 | 37 and 51 |
| 64 | 38 and 51 |
| 65 | 39 and 51 |
| 66 | 40 and 51 |
| 67 | 41 and 51 |
| 68 | 42 and 51 |
| 69 | 43 and 51 |
| 70 | 44 and 51 |

OVID/MEDLINE/EMBASE

<https://ovidsp.ovid.com/>

| 1 | aged.mp. [mp=ti, ab, hw, tn, ot, dm, mf, dv, kw, fx, dq, nm, kf, ox, px, rx, an, ui, sy] |
| --- | --- |
| 2 | elderly.mp. [mp=ti, ab, hw, tn, ot, dm, mf, dv, kw, fx, dq, nm, kf, ox, px, rx, an, ui, sy] |
| 3 | geriatric.mp. [mp=ti, ab, hw, tn, ot, dm, mf, dv, kw, fx, dq, nm, kf, ox, px, rx, an, ui, sy] |
| 4 | older person.mp. [mp=ti, ab, hw, tn, ot, dm, mf, dv, kw, fx, dq, nm, kf, ox, px, rx, an, ui, sy] |
| 5 | ADR prediction.mp. [mp=ti, ab, hw, tn, ot, dm, mf, dv, kw, fx, dq, nm, kf, ox, px, rx, an, ui, sy] |
| 6 | adverse drug reaction prediction.mp. [mp=ti, ab, hw, tn, ot, dm, mf, dv, kw, fx, dq, nm, kf, ox, px, rx, an, ui, sy] |
| 7 | adverse drug reaction detection.mp. [mp=ti, ab, hw, tn, ot, dm, mf, dv, kw, fx, dq, nm, kf, ox, px, rx, an, ui, sy] |
| 8 | BEERS criteria.mp. [mp=ti, ab, hw, tn, ot, dm, mf, dv, kw, fx, dq, nm, kf, ox, px, rx, an, ui, sy] |
| 9 | STOPP START.mp. [mp=ti, ab, hw, tn, ot, dm, mf, dv, kw, fx, dq, nm, kf, ox, px, rx, an, ui, sy] |
| 10 | gerontonet.mp. [mp=ti, ab, hw, tn, ot, dm, mf, dv, kw, fx, dq, nm, kf, ox, px, rx, an, ui, sy] |
| 11 | brighton adverse drug reaction.mp. [mp=ti, ab, hw, tn, ot, dm, mf, dv, kw, fx, dq, nm, kf, ox, px, rx, an, ui, sy] |
| 12 | WHO Uppsala.mp. [mp=ti, ab, hw, tn, ot, dm, mf, dv, kw, fx, dq, nm, kf, ox, px, rx, an, ui, sy] |
| 13 | Naranjo scale.mp. [mp=ti, ab, hw, tn, ot, dm, mf, dv, kw, fx, dq, nm, kf, ox, px, rx, an, ui, sy] |
| 14 | MERIS.mp. [mp=ti, ab, hw, tn, ot, dm, mf, dv, kw, fx, dq, nm, kf, ox, px, rx, an, ui, sy] |
| 15 | MEDCOINS score.mp. [mp=ti, ab, hw, tn, ot, dm, mf, dv, kw, fx, dq, nm, kf, ox, px, rx, an, ui, sy] |
| 16 | PADR-EC score.mp. [mp=ti, ab, hw, tn, ot, dm, mf, dv, kw, fx, dq, nm, kf, ox, px, rx, an, ui, sy] |
| 17 | Drug interaction probability scale.mp. [mp=ti, ab, hw, tn, ot, dm, mf, dv, kw, fx, dq, nm, kf, ox, px, rx, an, ui, sy] |
| 18 | or/1-4 |
| 19 | 5 and 18 |
| 20 | 6 and 18 |
| 21 | 7 and 18 |
| 22 | 8 and 18 |
| 23 | 9 and 18 |
| 24 | 10 and 18 |
| 25 | 11 and 18 |
| 26 | 12 and 18 |
| 27 | 13 and 18 |
| 28 | 14 and 18 |
| 29 | 15 and 18 |
| 30 | 16 and 18 |
| 31 | prevention.mp. [mp=ti, ab, hw, tn, ot, dm, mf, dv, kw, fx, dq, nm, kf, ox, px, rx, an, ui, sy] |
| 32 | reduce.mp. [mp=ti, ab, hw, tn, ot, dm, mf, dv, kw, fx, dq, nm, kf, ox, px, rx, an, ui, sy] |
| 33 | prevent.mp. [mp=ti, ab, hw, tn, ot, dm, mf, dv, kw, fx, dq, nm, kf, ox, px, rx, an, ui, sy] |
| 34 | avoid.mp. [mp=ti, ab, hw, tn, ot, dm, mf, dv, kw, fx, dq, nm, kf, ox, px, rx, an, ui, sy] |
| 35 | decrease.mp. [mp=ti, ab, hw, tn, ot, dm, mf, dv, kw, fx, dq, nm, kf, ox, px, rx, an, ui, sy] |
| 36 | "cut down".mp. [mp=ti, ab, hw, tn, ot, dm, mf, dv, kw, fx, dq, nm, kf, ox, px, rx, an, ui, sy] |
| 37 | ADR detection.mp. [mp=ti, ab, hw, tn, ot, dm, mf, dv, kw, fx, dq, nm, kf, ox, px, rx, an, ui, sy] |
| 38 | 18 and 37 |
| 39 | or/31-36 |
| 40 | 18 and 39 |
| 41 | 37 and 40 |
| 42 | 5 and 40 |
| 43 | 6 and 40 |

CINAHL

<https://www.ebsco.com/products/research-databases/cinahl-database>

| 1 | Aged |
| --- | --- |
| 2 | Elderly |
| 3 | Geriatric |
| 4 | “older person” |
| 5 | “older people” |
| 6 | ADR prediction |
| 7 | ADR detection |
| 8 | Adverse drug reaction prediction |
| 9 | Adverse drug reaction detection |
| 10 | ADR prediction tool |
| 11 | ADR detection tool |
| 12 | Adverse drug reaction prediction tool |
| 13 | Adverse drug reaction detection tool |
| 14 | BEERS criteria |
| 15 | STOPP/START |
| 16 | Gerontonet ADR Risk Score |
| 17 | Brighton adverse drug reaction risk |
| 18 | World health organization Uppsala monitoring centre |
| 19 | Naranjo scale |
| 20 | PADR-EC Score |
| 21 | Adverse drug reaction risk in older persons scale |
| 22 | MERIS |
| 23 | MEDCOINS Score |
| 24 | Drug interaction probability scale |
| 25 | or/1-5 |
| 26 | 6 and 25 |
| 27 | 7 and 25 |
| 28 | 8 and 25 |
| 29 | 9 and 25 |
| 30 | 10 and 25 |
| 31 | 11 and 25 |
| 32 | 12 and 25 |
| 33 | 13 and 25 |
| 34 | 14 and 25 |
| 35 | 15 and 25 |
| 36 | 16 and 25 |
| 37 | 17 and 25 |
| 38 | 18 and 25 |
| 39 | 19 and 25 |
| 40 | 20 and 25 |
| 41 | 21 and 25 |
| 42 | 22 and 25 |
| 43 | 23 and 25 |
| 44 | 24 and 25 |

PROQUEST

https://www.proquest.com/

| 1 | Elderly |
| --- | --- |
| 2 | Geriatric |
| 3 | ADR prediction |
| 4 | ADR detection |
| 5 | Adverse drug reaction prediction |
| 6 | Adverse drug reaction detection |
| 7 | ADR prediction tool |
| 8 | ADR detection tool |
| 9 | Adverse drug reaction prediction tool |
| 10 | Adverse drug reaction detection tool |
| 11 | BEERS criteria |
| 12 | STOPP/START |
| 13 | Gerontonet ADR Risk Score |
| 14 | Brighton adverse drug reaction risk |
| 15 | World health organization Uppsala monitoring centre |
| 16 | Naranjo scale |
| 17 | PADR-EC Score |
| 18 | Adverse drug reaction risk in older persons scale |
| 19 | MERIS |
| 20 | MEDCOINS Score |
| 21 | Drug interaction probability scale |
| 22 | 1 and 3 |
| 23 | 1 and 4 |
| 24 | 1 and 5 |
| 25 | 1 and 6 |
| 26 | 1 and 7 |
| 27 | 1 and 8 |
| 28 | 1 and 9 |
| 29 | 1 and 10 |
| 30 | 1 and 11 |
| 31 | 1 and 12 |
| 32 | 1 and 13 |
| 33 | 1 and 14 |
| 34 | 1 and 15 |
| 35 | 1 and 16 |
| 36 | 1 and 17 |
| 37 | 1 and 18 |
| 38 | 1 and 19 |
| 39 | 1 and 20 |
| 40 | 1 and 21 |
| 41 | 2 and 3 |
| 42 | 2 and 4 |
| 43 | 2 and 5 |
| 44 | 2 and 6 |
| 45 | 2 and 7 |
| 46 | 2 and 8 |
| 47 | 2 and 9 |
| 48 | 2 and 10 |
| 49 | 2 and 11 |
| 50 | 2 and 12 |
| 51 | 2 and 13 |
| 52 | 2 and 14 |
| 53 | 2 and 15 |
| 54 | 2 and 16 |
| 55 | 2 and 17 |
| 56 | 2 and 18 |
| 57 | 2 and 19 |
| 58 | 2 and 20 |
| 59 | 2 and 21 |
| 60 | Prevention |
| 61 | 22 and 60 |
| 62 | 23 and 60 |
| 63 | 24 and 60 |
| 64 | 25 and 60 |
| 65 | 26 and 60 |
| 66 | 27 and 60 |
| 67 | 28 and 60 |
| 68 | 29 and 60 |
| 69 | 30 and 60 |
| 70 | 31 and 60 |
| 71 | 32 and 60 |
| 72 | 33 and 60 |
| 73 | 34 and 60 |
| 74 | 35 and 60 |
| 75 | 36 and 60 |
| 76 | 37 and 60 |
| 77 | 38 and 60 |
| 78 | 39 and 60 |
| 79 | 40 and 60 |
| 80 | 41 and 60 |
| 81 | 42 and 60 |
| 82 | 43 and 60 |
| 83 | 44 and 60 |
| 84 | 45 and 60 |
| 85 | 46 and 60 |
| 86 | 47 and 60 |
| 87 | 48 and 60 |
| 88 | 49 and 60 |
| 89 | 50 and 60 |
| 90 | 51 and 60 |
| 91 | 52 and 60 |
| 92 | 53 and 60 |
| 93 | 54 and 60 |
| 94 | 55 and 60 |
| 95 | 56 and 60 |
| 96 | 57 and 60 |
| 97 | 58 and 60 |
| 98 | 59 and 60 |

This search strategy was translated as appropriate for the other databases
